# Extracellular RNA in kidney disease: moving slowly but surely from bench to bedside

**DOI:** 10.1042/CS20201092

**Published:** 2020-11-13

**Authors:** Robert W. Hunter, Neeraj Dhaun

**Affiliations:** Department of Renal Medicine, Royal Infirmary of Edinburgh, EH16 4SA and University/BHF Centre for Cardiovascular Science, The Queen’s Medical Research Institute, University of Edinburgh, EH16 4TJ, U.K.

**Keywords:** microRNA, renal physiology, therapeutics

## Abstract

We have known for just over a decade that functional RNA is shuttled between cells (*Nat. Cell Biol.* (2007) **9**, 654–659). In that short time, there have been countless reports of extracellular RNA (exRNA) and extracellular vesicles (EVs) participating in diverse biological processes in development (*Dev. Cell* (2017) **40**, 95–103), homoeostasis (*Nature* (2017) **542**, 450–455) and disease (*Nature* (2017) **546**, 498–503). Unsurprisingly – as these disciplines are still in their infancies – most of this work is still in the ‘discovery biology’ phase. However, exRNA and EVs show promise as disease biomarkers and could be harnessed in novel therapies.

The renal tubular epithelial cell (rTEC) has been well-characterised with respect to its ability to release and take up extracellular RNA (exRNA) and EVs [1,2]. We also know quite a lot about how these signals can reprogramme rTEC function. For example, Camussi and co-workers have demonstrated that extracellular vesicles (EVs) and exRNA from mesenchymal stem cells confer protection from acute kidney injury (AKI) in rodent models [3–6]. Similarly, hypoxic rTECs release EVs that can protect against AKI [7]; the complete mechanism is not known but in part relies on transfer of HIF-1α [8].

If we are ever going to bridge the chasm between bench and bedside, then we need to develop a thorough understanding of the molecular mechanisms mediating any therapeutic effect, and of the feasibility of delivering RNA-/vesicle-based reagents. In this context, the work reported by Yu and co-workers [9] is a small but significant step in the right direction. This group replicated the previous observation that EVs derived from hypoxic rTECs can protect against hypoxia-induced injury *in vitro*. In an attempt to determine the causative molecular mechanism, they used small RNA sequencing to detect microRNAs that were enriched in EVs from hypoxic (compared with normoxic) cells. They identified miR-20a-5p as one such miRNA and went on to show – using an agomir and antagomir approach – that this alone was sufficient to protect against hypoxia-induced defects in cell proliferation and mitochondrial function (Figure 1). Furthermore, they showed that the intravenous injection of a miR-20a-5p agomir could protect against tubular injury in a mouse ischaemia–reperfusion model.

**Figure 1:**
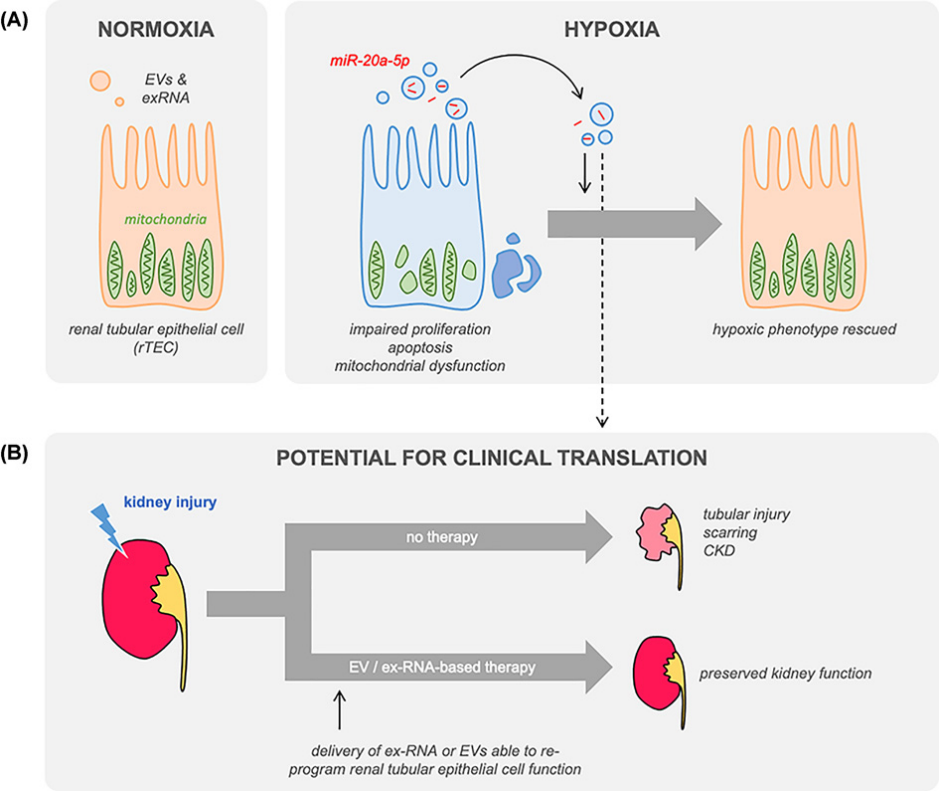
Protective role of microRNA in the kidney (**A**) **Conclusions from Yu and co-workers**. Under hypoxic conditions *in vitro*, renal tubular cells released EVs enriched in microRNAs that were predicted to target mitochondrial pathways, including miR-20a-5p [9]. These vesicles (or an miR-20a-5p agomir) ameliorated hypoxia-induced changes in mitochondrial function and cell proliferation. *In vivo*, the miR-20a-5p agomir protected rTECs from injury after ischaemia–reperfusion. (**B**) **Potential for clinical translation**. The present study adds to a literature supporting the principle that ex-RNA and EVs could provide effective therapies for kidney disease. ExRNA or EVs could be used to re-programme rTECs, ameliorating the atrophic and fibrotic sequelae of untreated kidney injury.

This reductionist approach – in which one or two critical miRNA species are identified and validated – has been widely followed by other researchers, e.g. [10–13]. The advantage is that one may be able to identify simple nucleic acid reagents that can be produced at scale and delivered safely and effectively to treat disease. For example, the miR-21 inhibitor RG-012 has entered clinical trials in Alport syndrome [14]. The disadvantage of this approach is that it sits at odds with what we know about EV and exRNA biology and is thus not able to answer questions of physiological relevance. We know that EVs contain a complex mix of multiple miRNAs (and other classes of small RNA) that are predicted to regulate thousands of target genes. It seems highly implausible that a single miRNA – at physiological concentrations – should be responsible for the bulk of this effect. Acknowledging that it may be impossible to decipher this complexity, alternative pragmatic strategies rely on attempts to produce therapeutic-grade artificial vesicles [15]. The vesicle ‘coat’ may prove to be critical: siRNA within a cell-derived vesicle is more effective at gene silencing than it is when delivered as naked nucleic acid or packaged within artificial lipid nanoparticles [16]. Ultimately, any significant breakthrough is likely to follow from a combination of both reductionist and pragmatic approaches.

The work reported by Yu and co-workers opens many questions regarding physiological relevance [9]. Does miR-20a-5p play a significant role in maintaining rTEC homoeostasis under physiological conditions? What role is played by the other miRNAs that are differentially regulated in hypoxia? Does the target cell differentiate between exogenous miR-20a-5p delivered in an EV and cell-endogenous miR-20-5p? Do the effects on mitochondrial function *cause* improved cellular health or are they merely associated?

There are also questions of clinical relevance. Is miR-20a-5p effective in AKI due to other (toxic, infective, inflammatory, vascular) causes? Does its delivery incite off-target toxicity? Is it effective at protecting rTECs if delivered hours or days after the injury? Would any effect translate into patient-centred clinical outcomes? Nevertheless, the present paper adds to the growing body of work suggesting that EV and exRNA-based therapeutics are possible in principle. It may be a small step on the road from bench to bedside, but every small step is welcome.

## Abbreviations

AKI: acute kidney injury
EV: extracellular vesicle
exRNA: extracellular RNA
rTEC: renal tubular epithelial cell
